# Correction to “RETRACTION: Clinical Effectiveness and Treatment Satisfaction Between Two Triple‐Therapy Regimens in Treating Neuropathic Pain: A Real World Data”

**DOI:** 10.1002/ibra.70023

**Published:** 2026-06-16

## Abstract

“RETRACTION: Clinical Effectiveness and Treatment Satisfaction Between Two Triple‐Therapy Regimens in Treating Neuropathic Pain: A Real World Data,” *Ibrain* 12, 1 (2026): 165. https://doi.org/10.1002/ibra.70012

The following section has been added to the retraction notice post publication:

Following publication of this retraction notice, co‑author Vennila Jaganathan contacted the journal to clarify that they were not aware of the issues leading to the retraction decision, that their contribution to the manuscript was limited to data analysis, and that, in their view, this contribution does not meet the criteria for authorship. They further stated that the decision to retract the article was made solely by the corresponding author Nithya Raju, who had not appropriately informed the co‐authors. Although communication regarding the retraction decision was sent to all co‑authors using the contact details provided at article submission, co‑author Vennila Jaganathan states that they were not informed of, and did not consent to, the retraction decision.

“RETRACTION: Clinical Effectiveness and Treatment Satisfaction Between Two Triple‐Therapy Regimens in Treating Neuropathic Pain: A Real World Data,” *Ibrain* 12, 1 (2026): 165. https://doi.org/10.1002/ibra.70012

The following section has been added to the retraction notice post publication:

Following publication of this retraction notice, co‑author Vennila Jaganathan contacted the journal to clarify that they were not aware of the issues leading to the retraction decision, that their contribution to the manuscript was limited to data analysis, and that, in their view, this contribution does not meet the criteria for authorship. They further stated that the decision to retract the article was made solely by the corresponding author Nithya Raju, who had not appropriately informed the co‐authors. Although communication regarding the retraction decision was sent to all co‑authors using the contact details provided at article submission, co‑author Vennila Jaganathan states that they were not informed of, and did not consent to, the retraction decision.

